# Clonal Extinction Drives Tumorigenesis

**DOI:** 10.3390/cancers15194761

**Published:** 2023-09-28

**Authors:** Adriana Amaro, Ulrich Pfeffer

**Affiliations:** Laboratory of Regulation of Gene Expression, IRCCS Ospedale Policlinico San Martino, 16132 Genova, Italy

Before a tumor is diagnosed and surgically removed, it has been growing for many months or even years [1]. The surgical sample, which can be analyzed by the pathologist or by molecular and cellular biologists, is the result of an evolutionary process that has selected the cellular clones that have eluded the self-regulation mechanism of normal cells and survived the organism’s attempts to eliminate them. Classically, we believed this evolution to occur stepwise by introducing mutations by replication errors or carcinogens or acquiring a mutator phenotype [2]. If the mutation yields a growth advantage under specific conditions and within a particular environment, the cells carrying it will grow faster than the other tumor cells. The iteration of this process is tumor evolution. Yet, we know from the evolution of species that the actual evolutionary process is much less linear, with phases of high genetic diversification and phases of stabilization of selected genotypes [3]. Analyses of many tumor clones from single patients have shown that tumor evolution follows a similar pattern [4,5,6]. Tumor initiation, likely determined by the loss of cell growth control and the presence of favorable environmental conditions, is followed by a period of genetic instability where high clonal diversity is generated, with each clone carrying a specific set of mutations. The selection of genetically more stable clones eventually leads to the clinically overt tumor. In simple words, tumorigenesis starts with a “big bang” [4] of genetic instability followed by the outgrowth of a minimal number of viable clones, essentially because the cell can tolerate only limited and precise genetic alterations without succumbing.

The real limit of tumor evolution studies is the impossibility of observing the process as it occurs. All of our understanding is derived from retrospective analyses of the mature tumor. In their recent article “Early clonal extinction in glioblastoma progression revealed by genetic barcoding” [7], the group led by Paolo Malatesta has undertaken an innovative approach to the study of tumor evolution by injecting embryonal mouse brains with retroviral expression constructs of the human platelet-derived growth factor B (PDGFβ), a known driver of glioblastoma pathogenesis. Preliminary experiments have shown that PDGFβ overexpression initiates tumorigenesis, and acquiring additional lesions determines full-blown growth of glioblastoma-like tumors in the adult mouse brain. The insertion of a bar code into the transcribed region of the PDGFβ cDNA, in a way that each brain cell receives a distinct label, has allowed for the clonal analysis from the beginning of tumorigenesis. These analyses essentially confirm what had been predicted using the big bang model based on the retrospective analysis of various tumor clones from a single patient. However, different from the prediction, barcoded tumors show a tendency towards the prevalence of a single clone that becomes particularly evident upon the transplantation of the tumor into the brains of syngeneic animals where a single clone formed a secondary glioma. Importantly, this differs from what was retrospectively observed by Sottoriva et al., who established the big bang model for colorectal cancer [4], as well as from the known clonal heterogeneity of human gliomas [8].

Yet, Ceresa and colleagues did not limit their analysis to the mere description of clonal heterogeneity. Their experimental approach also enabled them to analyze the transcriptional profile of specific clones using bulk and single-cell transcriptomics. By doing so, they tried to identify the molecular players that yield the growth advantage of surviving clones. Additional experiments showed that the clonal selection process continues even for late-stage gliomas when transferred to recipient mice. Analogous experiments with another driver of tumorigenesis yielded consistent results. Therefore, the single clone’s viability is not the only selection criterion. 

Computational simulation also excluded a statistical drift of the cell populations due to prevalent apoptosis and alterations of cell cycle dynamics. Clonal competition, a concept introduced by Moreno and coworkers [9], was the only explanation that resisted simulation, and transcriptional analyses of the successful clones concordantly showed that activation of the c-MYC pathway makes the game consistent with what we know about this driver of cell cycle progression, apoptosis, cellular transformation [10] and cell competition [11].

However, these results starkly contrast with what we know about molecular and cellular heterogeneity of human gliomas that may contain different subtypes within the same tumor mass [12]. How can the results of the hPDGFβ model be reconciled with the known heterogeneity of human gliomas?

− The clonal extinction observed might be typical for the two initiating molecular lesions studied (PDGFβ and epidermal growth factor receptor variant III [EGFRvIII]) but not for other events. This can only be ruled out by experiments with other glioma drivers, such as Isocitrate Dehydrogenase (NADP(+)) 1 (IDH1) [13].
− Copy number alterations and somatic mutations generate clonal heterogeneity, but this has not been analyzed by Ceresa et al., who show that the transcriptional profile of different clones is not dramatically different yet evidently enough to alter the c-Myc pathway. Amplification of the locus containing c-Myc that occurs in many human tumors [14] might contribute to overexpression of the gene and the consequent competition advantage.
− Phenotypic plasticity [15], detectable at the protein level, could generate clonal heterogeneity that escaped the molecular analyses performed.
− Human tumors develop on a heterogeneous cellular background, given that the cells of an adult human already contain many somatic mutations before the tumor-initiating mutation occurs [16]. This is, however, important only for tumors generated using field cancerization [17], which, to the best of our knowledge, has not been described for gliomas.
− The experimental tumors have been grown in immunocompetent mice. Acquisition of additional somatic mutations early during tumorigenesis might, in part, be recognized by the immune system and lead to eliminating the subclones expressing such neoantigens. Immunoediting might thus contribute to clonal extinction [18].
− The difference between men and mice might account for different levels of tumor heterogeneity. Murine tumors are much smaller than human tumors (just like the respective hosts), but the cells that form the tumor are of similar size [19]. Consequently, murine tumors contain much fewer cells than human tumors, with consequently lower chances of heterogeneity. In large human tumors, a clone with relatively little cellular competition potential can grow independently from a distant, more competitive clone simply because the former is not exposed to and, therefore, not in competition with the latter. To perform the experiments in a reasonable time, the authors had to choose a highly penetrant tumor initiator, hPDGF-β, that led to a fulminant tumor development with approximately half of the animals carrying life-threatening tumors 50 days after inoculation of the retroviral construct. Human tumors develop much more slowly, leaving more time for the evolution of stable clones.

The best explanation for the results observed in the light of tumor evolution models is the application of models of phylogenetic evolution of the species that rely on punctuate equilibrium but also contemplate the continuous birth and death (by competition) of new species, similar to clonal fluctuations in cancer cell populations. Tumors continuously evolve, driven by cell competition and alterations of the environment brought upon by the immune response and cancer treatment.

Much work must be performed to deeply understand to which extent experimental tumors reflect the human situation. Anyway, the work by Ceresa et al. highlights the importance of cellular competition in tumor evolution. Why is this important to know? Understanding tumor evolution from the molecular and cellular point of view is necessary to understand the dynamics of the response to the treatment of a moving (evolving) target [20], and cellular competition might present additional targets for tumor therapy [9]. If we understood how cancer cells lose in the survival race, we would be able to exploit competition when designing therapies. 
